# Hyperthermal Medication*Abstract of a paper read before the Society of Comparative Pathology of France, and published in *La Revue de Pathologie Comparee*, January, 1908.

**Published:** 1910-04

**Authors:** Barthe De Sandfort

**Affiliations:** Paris, France, Surgeon of the French Navy, First Class, Retired; Fellow de la Societe de Medicine et Chirurgie de Paris; Fellow de L’Institut de Pathologie Comparee de France, etc.


### Hyperthermal Medication.*

BY BARTHE DE SANDFORT, M. D., PARIS, FRANCE,

Surgeon of the French Navy, First Class, Retired; Fellow de la Societe de Medicine et Chirurgie de Paris; Fellow de L'Institut de Pathologie Comparee de France, etc.

What is meant by hyperthermal medication? The word thermal at once reminds one of mineral springs, and in using it the intention was to indicate the precise starting point of this article which, in a measure, is the continuation of my former studies at the Mud Baths in Dax, France.

---

*Abstract of a paper read before the Society of Comparative Pathology of France, and published in *La Revue de Pathologie Comparee*, January, 1908.

In thermal medication the highest temperature tolerated in the shape of baths or tropical applications (mineral mud) is never higher than 113°, and we know that Professor Reclus, one of the most eminent protagonists of hot water, has often expressed his regret at not being able to exceed 122° in the hot water dressings from which he derived such marvelous results.

The extreme heat limit, therefore, in thermal medication is 122°; beyond that hyperthermal medication commences. Employed already in the times of remote antiquity in the vaporia and tepidoria of the ancients, it has today acquired novel attractions, since the firewood of by-gone days and the alcohol of our forefathers have been replaced by gas and electricity; and especially since those famous hot air boxes, manufactured abroad and imported here, appeal to the patient by reason of their magnificent high degrees of heat. It seems that the 300° to 392° of superheated air which they achieve exert a mysterious effect. I have neither the time nor intention to show how old this alleged novelty is, and it will suffice to state that this cumbersome heating apparatus can not be applied in the ordinary conditions of life.

If it is possible to apply air heated up to 200° to the skin without injuring it, it is due to the well known mechanical effects of perspiration, and the treated part is merely subjected to circumambient heat. In those boxes as well as in steam baths the effect resolves itself into a peripheral stimulation at a distance from the exposed region, which is no greater than if humidity were absent. Quite different is the action of hot epithems, such as sand bags, thermal mud baths, hot compresses or cataplasms, because here there is direct contact between the generated heat and the tissues, and since up to now the medium was always aqueous, the limit of tolerance of the skin was necessarily restricted to 122°.

Between hyperthermalization by radiation and that by contact it has seemed possible to us to establish a link, for our method has little resemblance to the two above referred to; it is a third modality of heat which in spite of direct contact between the generated heat and the tissues permits the latter to be subject to hyperthermic temperatures. As will be seen, it is sufficient to replace the hydrous element of ordinary epithems by a strictly anhydrous substance, which brings us near hot dry air. After many studies and experiments we have decided upon a mixture of various kinds of waxes and resins (kero-resin) to which we have given the name of "Hyperthermine."

This mixture, which in its cold state is solid, becomes fluid when subjected to heat either direct or in a water bath, and it can be applied like an epithem or by spraying it like water on the tissues themselves.

Hyperthermine is characterized by three distinct properties:

1. It is tolerated without causing changes, at temperatures varying between 180° and 220°, according to whether it is applied to a corium or not.

2. It keeps up the temperature of the application at a much higher degree and for an infinitely longer time, than anything observed before.

3. It contracts in cooling; and it is this property, by far the most interesting, which gives it a unique place and really constitutes the originality of the method, which I will now proceed to explain.

First.—The tolerance of the tissues to the influence of Hyperthermine is certainly what strikes one most at first; you may dip the finger into the substance at 280° without burning; you may apply to the skin successive layers of liquefied Hyperthermine which, when tested by the thermometer, would register 180° to 220°. This would be astonishing but for the well known phenomenon of calefaction, and it will be understood that the physiologic and therapeutic effects of such a heat wave must be considerable.

Second.—The equally important question as to whether this heat is retained can be easily decided by putting into a test glass 125 grams of hot water with 7 grams of cotton wool, which is the proportion of water absorbed by a pad of cotton wool; and into another similar glass 125 grams of hot Hyperthermine together with 7 grams of cotton wool. Let the temperature of each glass, as registered by the thermometer, be 122° (the maximum tolerance for a cotton pad) and at the end of twenty-five minutes it will be seen that the temperature of the water has dropped to below 104°, which means that it has no longer any thermic effect, while the Hyperthermine in the other glass will have a temperature of about 113° after a lapse of two and one-half hours.

Thus, it is an established fact that a dressing made with Hyperthermine will retain a temperature of over 104° longer than one made with water, and also that we may bandage the skin with this substance at a very much greater temperature without causing burns. This then is certainly a hyperthermic application.

Third.—But these properties which experience has shown to exist would, in spite of their practical advantages, not have appealed to me sufficiently to claim superiority for them over ordinary local calefaction but for the additional advantage of contractility. A limb enveloped in successive layers of Hyperthermine and cotton, forming a thickness of about 3 cm., soon manifests a distinct sensation of constriction, and this results from the contraction taking place during the solidification of the substance while cooling down from its initial 180° or 220° to 110° or 115°, which it will now retain in its lowest layer.

There is thus greater heat, combined with the advantages of a slow, gentle, uniformly progressive compression, the mechanical importance of which in articular, glandular, testicular and especially phlebitic and varicose engorgements is too evident to require argument. Its physiological importance is equally great, and it assures for Hyperthermine both a novel and exceptional position.

Up to the present the study of hot applications and their effects on the circulation and the other functions has never taken into account anything except temperature. Now, however, is added automatic compression which, in accordance with the effects observed, entitles us to make the following aphorismic statement: The heat formerly utilized in therapeutics, in whatever form, was a "passive" heat, if we may be pardoned for so calling it, while now we possess an "active" heat. As a matter of fact, a cotton pad or a hot compress are only vehicles of heat and nothing else, as they can not of themselves exert a mechanical effect, while Hyperthermine, besides being a vehicle of heat, acts at the same time as a mechanical agent of compression.

Ordinary applications of heat can only produce a passive dilatation of the peripheral net, which, after they have ceased to act, will contract progressively, but passively; in our method the action of Hyperthermine produces intense hyperemia of the skin, which means that there is not only dilatation of the peripheral net, as in the other process, but also that under the influence of its contractility the blood of the peripheral net is mechanically returned by the continued pressure towards the deeper net, so that the local circulation, the circulatory elasticity, is powerfully activated.

But this contractility has other very interesting effects, because Hyperthermine is also used in the dressing of wounds. Having, by heating, become as fluid as water, it may be handled exactly like it, which means it may be injected into a cavity which it will fill precisely as would an injection of water. After the tissues have thus been bathed for a few minutes in this strictly aseptic fluid at a temperature in the vicinity of 200°, an intense stimulation of the blood supplying this region will have been produced, thus increasing phagocytosis. Soon, however, the substance will cool down to about body temperature; it contracts, occupying less space than before, and as it has become moulded upon the walls of the wound, it forms a real wax cone, leaving between it and the traumatic walls a space for drainage, as the cone will not adhere to the tissues, but will remain suspended in the middle of the cavity. The presence of this internal mould has two effects; in the first place, as the Hyperthermine solidifies it engulfs, and, therefore, isolates, all foreign matter, such as detritus and necrosed matter which may be infecting the wound. Next, owing to its shape, it forces the granulating ends to follow each other with uniform intensity over the entire internal surface of the wound. This impulse, which is likewise brought to bear mechanically on the physiologic action, regulates the progress of cicatrization, forces the successive stratifications of fibrous tissue to proceed uniformly through the wound from depth to surface, and thus explains the suppleness of the scar obtained.

Thus, Hyperthermine is a physico-mechanical means to obtain automatically (by elevated temperature) complete asepsis, cleansing and regularity of the scars, without any intervention on the part of the operator.

It can easily be understood what the therapeutic consequences of the physical properties will be as we have just enumerated.

Hyperthermine will give valuable aid to the physician, the surgeon or the veterinary; in fact the field is so large that we can perfectly understand the suspicion with which it is sometimes received. If, however, it is looked upon not as a medicament, *as it does not contain any medicinal substance whatever, but as a vehicle of heat, and as a mechanical means of compression and disinfection,* it represents a purely physical agent, and as such its use should not be confined to narrow limits.

Heat has been applied for all times by physicians, surgeons and veterinaries. We offer you a new method of localizing intense and painless heat in an affected region, and it makes no difference whether this agent can be used for a number of various purposes, so long as it is practically useful and applicable in all possible conditions.

NOTE.—Hyperthermine has been known for the past ten years in France as L'Ambrine, and the original French literature refers to the product under the latter name. As Hyperthermine has a definite meaning, the remedial agent will be known in this country only as Hyperthermine. It is manufactured in France, and the Thermo-Chemicals Company, 6 Cliff Street, New York, is the exclusive selling agent for the United States, Canada and Mexico.

